# *Trans*-chalcone increases p53 activity via DNAJB1/HSP40 induction and CRM1 inhibition

**DOI:** 10.1371/journal.pone.0202263

**Published:** 2018-08-17

**Authors:** Gabriel Silva, Mozart Marins, Nadda Chaichanasak, Yongdae Yoon, Ana Lúcia Fachin, Vitor Caressato Pinhanelli, Luis Octávio Regasini, Mariana Bastos dos Santos, Gabriela Miranda Ayusso, Beatriz de Carvalho Marques, Wells W. Wu, Je-Nie Phue, Rong-Fong Shen, Seung Joon Baek

**Affiliations:** 1 Biotechnology Unit, University of Ribeirão Preto, Ribeirão Preto, São Paulo, Brazil; 2 Medicine School, University of Ribeirão Preto, Ribeirão Preto, São Paulo, Brazil; 3 Laboratory of Signal Transduction, College of Veterinary Medicine and Research Institute for Veterinary Science, Seoul National University, Seoul, Republic of Korea; 4 Department of Chemistry and Environmental Chemistry, São Paulo State University (UNESP), São Paulo, Brazil; 5 Facility for Biotechnology Resources, CBER, Food and Drug Administration, Silver Spring, Maryland, United States of America; Augusta University, UNITED STATES

## Abstract

Naturally-occurring chalcones and synthetic chalcone analogues have been demonstrated to have many biological effects, including anti-inflammatory, anti-malarial, anti-fungal, and anti-oxidant/anti-cancerous activities. Compared to other chalcones, *trans*-chalcone exhibits superior inhibitory activity in cancer cell growth as shown via *in vitro* assays, and exerts anti-cancerous effects via the activation of the p53 tumor suppressor protein. Thus, characterization of the specific mechanisms, by which *trans*-chalcone activates p53, can aid development of new chemotherapeutic drugs that can be used individually or synergistically with other drugs. In this report, we found that *trans*-chalcone modulates many p53 target genes, HSP40 being the most induced gene in the RNA-Seq data using *trans*-chalcone-treated cells. CRM1 is also inhibited by *trans*-chalcone, resulting in the accumulation of p53 and other tumor suppressor proteins in the nucleus. Similar effects were seen using *trans*-chalcone derivatives. Overall, *trans*-chalcone could provide a strong foundation for the development of chalcone-based anti-cancer drugs.

## Introduction

Chalcones are receiving lots of attention recently because they exhibit diverse biological activities. They can exist in two major isomeric forms, *cis* and *trans*; the *trans*-chalcone (TChal) is considered to be more thermodynamically stable [1]. TChal has been shown to exert cytotoxic activity against many cancer cells via multiple mechanisms including cell cycle disruption, angiogenesis inhibition, tubulin polymerization inhibition, apoptosis induction, and blockade of the NF-κB signaling pathway [2]. Moreover, it has also been identified as an inhibitor of topo 2A (Top2A) [3], which is a target of many anti-cancer drugs. Molecules like TChal have relatively low redox potentials and have a greater probability of undergoing electron transfer reactions due to conjugated double bonds. This property of TChal along with lipophilicity enhances its biological activity. Thus, TChal is found to be a suitable precursor for the synthesis of various heterocyclic derivatives such as pyrimidine, imidazole, thiazine, thiazole, quinolone, oxazole, benzopyran, indole, and imidazolone [4].

Regulation of p53 primarily takes place at the protein level within a regulatory network, where p53 is polyubiquitylated by its inhibitor MDM2 and subsequently degraded by the proteasome machinery [5, 6]. Stability and activation of p53 can be enhanced by blocking its MDM2-mediated ubiquitination via the interaction of MDM2 with proteins such as DNAJB1, a member of the HSP40 family [7]. Another p53 regulation mechanism involves the nuclear exporter system. Inhibition of the nuclear exporter accumulates p53 proteins in the nucleus and triggers the activation of various p53 target genes such as p21, 14-3-3, growth arrest and DNA-damage-inducible 45, Bax, Bid, Puma, Noxa, FAS, PRSP, and APAF-1, resulting in cell-cycle arrest, senescence, and/or apoptosis [8].

CRM1 (also known as Exportin1) is a nuclear exporter involved in the active transport of tumor suppressors; its function is altered in cancer due to increased expression and overactive transportation [9]. CRM1 could be considered as a promising therapeutic target for anticancer drug development because the overexpression of CRM1 has been correlated with poor prognosis of patients in several cancers. CRM1 interacts with p53, and this inhibition of CRM1 results in the accumulation of p53 in the nucleus [9].

Increasing evidence suggests that stabilization of mutant p53 in tumors is crucial for oncogenic activities, whereas depletion of mutant p53 attenuates the malignant properties of cancer cells. Interestingly, the rescue of p53 activity in mutant p53 cancer cells has been shown to induce regression of various types of tumors such as soft tissue sarcomas and hepatocellular carcinomas, with the added advantage of normal tissues not being significantly affected by the genetic re-establishment of p53 [10–13]. Together with p53 stabilization, p53 functional restoration via specific compounds promises to be an interesting strategy in developing new cancer therapies [14–16].

In this report, we found that TChal could enhance the expression of HSP40 at the transcriptional level and inhibit CRM1 at the protein level, thereby enhancing p53 accumulation in the nucleus. These results highlight the potential of exploring TChal as a versatile molecule for studying cancer pathways and developing anti-cancer drugs based on p53 activation.

## Materials and methods

### Cell lines and reagents

Human cancer cell lines, U2OS, HCT-116, SJSA-1, and FaDu, were purchased from the American Type Culture Collection (ATCC, Manassas, VA, USA). These cells were tested by ATCC for post-freeze viability, growth properties, morphology, mycoplasma contamination, species determination (cytochrome c oxidase I assay and short tandem repeat analysis), sterility test and human pathogenic virus testing. The cell-lines were straightaway resuscitated upon receiving (Mar 2015) and frozen in aliquots in liquid nitrogen, and each frozen vial was cultured within six months. U2OS and HCT-116 were cultured in McCoy’s 5A medium. FaDu and SJSA-1 cells were maintained in EMEM and RPMI-1640 medium, respectively. All culture media were supplemented with 10% FBS, 100 U/mL penicillin, and 100 mg/mL streptomycin, while all culture cells were maintained at 37°C under a humidified atmosphere containing 5% CO_2_. *Trans*-chalcone was purchased from Alfa Aesar (Ward Hill, MA, USA). The following antibodies were purchased from Santa Cruz Biotechnology (Santa Cruz, CA, USA): anti-p53, anti-HSP40, anti-ATF-3, anti-CRM1, anti-Lamin A/C, anti-β-Actin. Anti-tubulin α and anti-Flag antibodies were purchased from Thermo Scientific (Rockford, IL, USA). Anti-PARP and anti-Cleaved Caspase-3 were purchased from Cell Signaling (Danvers, MA, USA).

### Synthesis of chalcone derivatives

Chalcones were synthesized by the Claisen-Schmidt condensation reaction between acetophenone derivatives and aromatic aldehydes using green solvents and catalysts. The structure of the compounds was confirmed by ^1^H and ^13^C Nuclear Magnetic Resonance (NMR) and bi-dimensional NMR techniques. Purity grade of the compounds were determined by High Performance Liquid Chromatography with Ultraviolet and Mass Spectrometry detection. Samples with a purity ≥ 95% were used for the bioassays.

### RNA isolation and Next Generation Sequencing (NGS)

U2OS cells were grown and treated with or without 50 μM of *trans*-chalcone in serum-free media for 24 h. Total RNA was isolated using the GeneJET RNA Purification Kit (Thermo Scientific) according to the manufacturer’s protocol. For RNA-Seq library preparation, an Illumina TruSeq RNA kit (V2; San Diego, CA, USA) was utilized and subsequently, paired-end sequencing of the RNA-Seq libraries (100 cycles) were realized on an Illumina HiSeq 2500, according to the manufacturer’s standard protocol. The sequence reads obtained from the sequencer were processed and analyzed as previously described [17]. Based in the distribution of the FPKM value of the transcripts, a list of expressed genes was defined to include only transcripts with an average FPKM > 1, in at least one of the treatments [18]. Using these transcripts, a list of differentially expressed genes (DEG) in response to TChal treatment was generated to include genes with fold-change (FC) > 2.0, using a threshold p-value of 0.001. Gene Ontology (GO) and Kyoto Encyclopedia of Genes and Genomes (KEGG) pathway enrichment analyses of the DEGs between trans-chalcone treated and untreated U2OS cells were performed using the “Gene List Analysis” (http://pantherdb.org/tools/index.jsp) and KEGG Mapper-Search Pathway” (http://www.genome.jp/kegg/tool/map_pathway1.html). A p-value < 0.05 was considered to indicate a statistically significant difference and was set as the cut-off. To examine the differential expression data obtained with RNA-Seq, Ingenuity Pathway Analysis software (IPA) (Qiagen Bioinformatics, https://www.qiagenbioinformatics.com/products/ingenuity-pathway-analysis/) was used. Different tools available on IPA, such as Canonical Pathways Analysis, Downstream Effects Analysis, and Molecule Activity Predictor were applied to predict the important biological downstream effects that were impacted by differential gene expression patterns found in the RNA-Seq dataset. The fastq datasets are available in the NCBI repository BioProject ID PRJNA480769 (https://www.ncbi.nlm.nih.gov/Traces/study/?acc=SRP153139).

### Reverse transcription-polymerase chain reaction (RT-PCR)

Isolated total RNA was treated with Optizyme Recombinant DNase I (Fisher Scientific, Pittsburgh, PA) and reverse-transcribed using a Verso cDNA Synthesis Kit (Thermo Scientific). PCR was carried out using the GoTaq^®^ Green Master Mix (Promega, Madison, WI, USA) with human primers as follows: *p21* Fwd 5'-gcgactgtgatgcgctaat-3' and Rev 5'-tagggcttcctcttggagaa-3', *Gadd45A* Fwd 5'-ggaggaagtgctcagcaaag-3' and Rev 5'-tcccggcaaaaacaaataag-3', *HSP40* Fwd 5'-tcactttgtgggtcacactctt-3' and Rev 5'-tccacagtgctcctgtaatcag-3', *ATF3* Fwd 5'-gtttgaggattttgctaacctgac-3' and Rev 5'-agctgcaatcttatttctttctcgt-3', and *GAPDH* Fwd 5'-gaccacagtccatgccatcact-3' and Rev 5'-tccaccaccctgttgctgtag-3'. The thermal cycling conditions were as follows: initial denaturation at 94 °C for 2 min, followed by 22 cycles of 94 °C for 30 sec, 60 °C for 30 sec, 72 °C for 45 sec, and final extension at 72 °C for 5 min. The PCR products were electrophoresed on 1.5% agarose gel and visualized under UV light.

### DNA constructs and transfection

The pHSP40-Luc construct containing human HSP40 promoter was generously provided by Dr. Debashis Mitra (Pune University, India). The promoters, pMDM2-Luc [19] and pATF3-Luc [20], were previously described. The expression vectors Flag-p53 and Flag-CRM1 were purchased from OriGene (Rockville, MD, USA). Transient transfections were carried out using the PolyJet Transfection Reagent (SignaGen, Gaithersburg, MD, USA) according to the manufacturer’s protocol.

### RNA interference

The *HSP40* siRNA was purchased from Santa Cruz Biotechnology and control siRNA was from Ambion (Austin, TX, USA). U2OS cells were transfected with 30 nM of *HSP40* or control siRNAs using PepMute^™^ siRNA Transfection Reagent (SignaGen). After transfection for 24 h, cells were treated as indicated, following which protein samples or RNA were obtained and subjected to western blot or RT-PCR analyses, respectively.

### Western blotting and subcellular fractionation

Cells were grown to 80% confluence in 60-mm dishes and treated as indicated in serum-free media. Protein lysates were obtained using RIPA buffer supplemented with proteinase inhibitors, separated on 12% SDS-PAGE gels, and transferred onto nitrocellulose membranes (Pall Corporation, Pensacola, FL). The membranes were blocked with TBST buffer (25 mM Tris, 3 mM KCI, 0.14 M NaCl, 0.05% Tween-20) containing 5% non-fat milk at room temperature for 1 h, and incubated overnight in TBST-5% non-fat milk containing primary antibodies at 4 °C. After three washes with TBST, the membranes were incubated with horseradish peroxidase-conjugated secondary antibodies for 1 h and then washed several times. Protein expression was detected by chemiluminescence using the ECL Western Blotting Detection Reagent (Amersham Biosciences, Piscataway, NJ, USA) in the luminescence analyzer, LAS4000 (Fujifilm Medical Systems, Stamford, CT). For subcellular fractionation, the Nuclear Extract Kit (Active Motif, Carlsbad, CA, USA) was used according to the manufacturer’s protocol. Subsequently, nuclear and cytoplasmic fractions were subjected to western blot analysis as described above.

### Immunoprecipitation

For immunoprecipitation analysis, 300 μg of protein lysates, collected using modified RIPA buffer (25 mM Tris-Cl (pH7.4), 150 mM NaCl, 1% NP-40, and 5% glycerol), were incubated with 2 μg of primary antibodies for 2 h at 4 °C on a rotating platform, followed by an overnight incubation in 20 μL of protein A/G PLUS-agarose (Santa Cruz Biotechnology). Immunoprecipitated samples were collected via centrifugation at 2,000 ×*g* for 5 min at 4 °C. After washing 10 times with ice-cold PBS, the pellets were resuspended with 40 μL of 4× SDS-PAGE sample loading buffer and heated at 95 °C for 5 min. Immunoprecipitated samples were analyzed via western blot as described above.

### Immobilization on magnetic beads and precipitation

*Trans*-chalcone was immobilized on SiMAG-Amine magnetic beads (Chemicell, Berlin, Germany), according to the manufacturer’s protocol. Briefly, 1 mg of *trans*-chalcone was immobilized on 10 mg of magnetic beads via the Mannich reaction, in which the *trans*-chalcone aromatic ring was covalently coupled to terminal amine groups of the magnetic beads by condensation with formaldehyde. The concentration of *trans*-chalcone in the supernatant (unbound *trans*-chalcone) was measured via UV-Vis spectroscopy. The amount of unbound *trans*-chalcone was subtracted from the initial amount of *trans*-chalcone used in the reaction to measure the quantity of *trans*-chalcone immobilized on the magnetic beads. For precipitation using magnetic beads, U2OS cells were transfected with a CRM1 expression vector and then cell lysates were obtained in TBST. Next, free-magnetic beads or beads bound with *trans*-chalcone were incubated overnight with cell lysates (1 mg) at 4 °C. After several washes, the beads were collected magnetically, resuspended in a sample buffer, and then subjected to western blot analysis.

### Immunofluorescence

For immunofluorescence, U2OS cells were seeded on a 12-well plate. Cells were then transfected with an expression vector containing a NAG-1/GFP fusion gene. After transfection, U2OS cells were treated with 50 μM of *trans*-chalcone for 24 h. Next, cells were washed thrice with PBS and then fixed with 1 mL of 4% paraformaldehyde containing 300 nM of DAPI (Roche Diagnostics, Indianapolis, IN, USA) for 10 min in the dark. After 4 PBS washes, fluorescence was observed at 100× magnification using the EVOS FL Auto Cell Imaging System (Invitrogen, Carlsbad, CA, USA).

### Luciferase assay

Cells were grown in 60-mm dishes to 80% confluence. Different luciferase promoter constructs were co-transfected with the pRL-null vector for 24 h. After the transfection, cells were treated for 24 h in serum-free media, and then harvested using 1× passive lysis buffer (Promega, Madison, WI, USA). Luciferase activity was measured and normalized to the pRL-null luciferase activity using a Dual-Glo^®^ Luciferase Assay Kit (Promega).

### Caspase-3/7 enzyme activity

The enzymatic activity of caspase-3/7 was analyzed using the Apo-ONE^®^ Homogeneous Caspase-Glo 3/7 Assay (Promega). U2OS cells, treated as indicated in media with 10% FBS for 24 h, were harvested in passive lysis buffer (Promega) containing protease and phosphatase inhibitors, and then lysed by sonication. Cell lysates from U2OS cells (50 μg protein) were mixed with the same volume of caspase-Glo 3/7 reagent in black-walled 96-well plates and incubated at room temperature in the dark for 1 h. Luminescence was measured using a FLX800 microplate reader (BioTek Instruments, Winooski, VT, USA).

### Statistical analysis

Statistical analysis was carried out using the Student’s unpaired *t*-test. Results were considered statistically significant at *, P < 0.05; **, P < 0.01; and ***, P < 0.001.

## Results

### *Trans*-chalcone (TChal) induces the p53 signaling pathway at the transcriptional level

**Fig 1:**
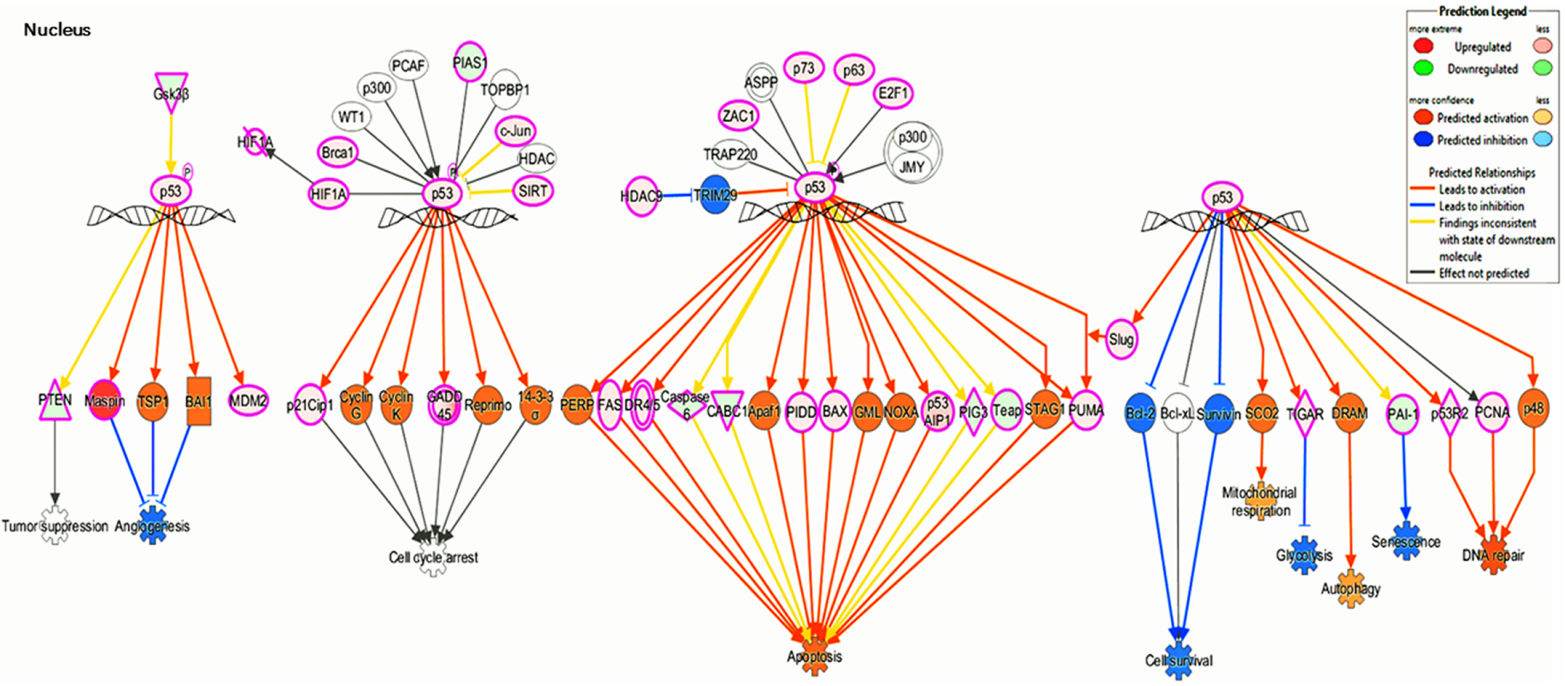
TChal activated the p53 signaling pathway at the transcriptional level. U2OS cells were treated with 50 μM of DMSO or TChal for 24 h. The RNA was isolated, and then RNA-Seq analysis was performed. The Ingenuity Pathway Analysis (IPA) software was applied to analyze the differential gene expression data. The p53 signaling pathway was activated by TChal, and the downstream effects of p53 activation induced by TChal was determined using the tools available on the IPA.

The p53 tumor suppressor protein is one of the most important transcription factors that controls many genes related to metabolic diseases. We previously reported that TChal induced p53 expression at the protein level in a proteasome-dependent manner, and subsequently increased its target genes, such as *p21* and *Gadd45a* [21]. To further investigate how TChal-induced p53 expression altered transcriptomes in p53 wild-type cells, RNA-Seq analysis was performed. U2OS osteosarcoma cells (p53 wild-type cells) were treated with 50 μM of TChal for 24 h (optimized dose and time for p53 induction by TChal) [21], and RNAs were isolated and subjected to RNA-Seq. The filtering of the FPKM values of the individual transcripts resulted in the assignment of 13,058 genes and a set of 4359 of these transcripts showed significant differential expression between control and TChal treated U2OS cells (p-value < 0.001). In order to capture the most significantly modulated genes by TChal treatment, a fold change threshold (2,0) was used to generate a list of 3233 DEGs, with 1521 up-regulated and 1712 down-regulated. The analysis of enriched KEGG and Panther pathways using the set of up-regulated DEGs showed genes associated to spliceosome, cell cycle, p53 pathways and apoptosis. Among the pathways regulated by TChal, p53 signaling pathway was identified as one of the most highly affected signaling pathways (Table 1). The down-regulated DEGs also were enriched for cancer related pathways such as Wnt/β-Cadherin signaling pathways. Among the up-regulated genes in the TChal-treated sample, *HSP40* and *ATF3* genes were highly induced (log_2_FC 5.82 and 4.72 respectively). These genes are involved in enhancing stability and activation of p53 by blocking its MDM2-mediated ubiquitination through the interaction of MDM2 [7, 22]. Moreover, based on the gene expression changes, alterations in cellular processes such as apoptosis, DNA repair, senescence, cell survival, and angiogenesis were predicted to be caused by TChal using Molecule Activity Predictor tool from IPA (Fig 1).

**Table 1:** Modulation of p53-related genes (transcriptional regulated or protein interaction) by trans-chalcone.

Gene Symbol	Official Full Name	Fold
**DNAJB1**	DnaJ heat shock protein family (Hsp40) member B1	**58.6**
**ATF3**	Activating transcription factor 3	**25.9**
**TP53AIP1**	Tumor protein p53 regulated apoptosis inducing protein 1	**17.9**
**PLK2**	Polo like kinase 2	**17.7**
**p21**	Cyclin-dependent kinase inhibitor 1A (CDKN1A)	**9.9**
**SERPINE1**	Serpin family E member 1	**7.3**
**FAS**	Fas cell surface death receptor	**6.1**
**BTG2**	BTG anti-proliferation factor 2	**5.2**
**BTG3**	BTG anti-proliferation factor 3	**3.3**
**p19**	Cyclin-dependent kinase inhibitor 2D (CDKN2D)	**2.8**
**DNAJC7**	DnaJ heat shock protein family (Hsp40) member C7	**2.8**
**RRM2B**	Ribonucleotide reductase regulatory TP53 inducible subunit M2B	**2.8**
**PUMA**	BCL2 binding component 3 (BBC3)	**2.7**
**PIDD**	p53-induced death domain protein 1	**2.6**
**TP63**	Tumor protein p63	**2.3**
**TP73**	Tumor protein p73	**2.3**
**DR5**	TNF receptor superfamily member 10b (TNFRSF10B)	**2.3**
**NOXA**	Phorbol-12-myristate-13-acetate-induced protein 1 (PMAIP1)	**2.1**
**BAX**	BCL2 associated X, apoptosis regulator	**1.7**
**ITGB1**	Integrin subunit beta 1	**-2.0**
**GPRC5A**	G protein-coupled receptor class C group 5 member A	**-3.8**
**GAS6**	Growth arrest specific 6	**-4.3**
**MN1**	MN1 proto-oncogene, transcriptional regulator	**-12.5**

### *Trans*-chalcone increases HSP40 and ATF3 expression in cancer cells

**Fig 2:**
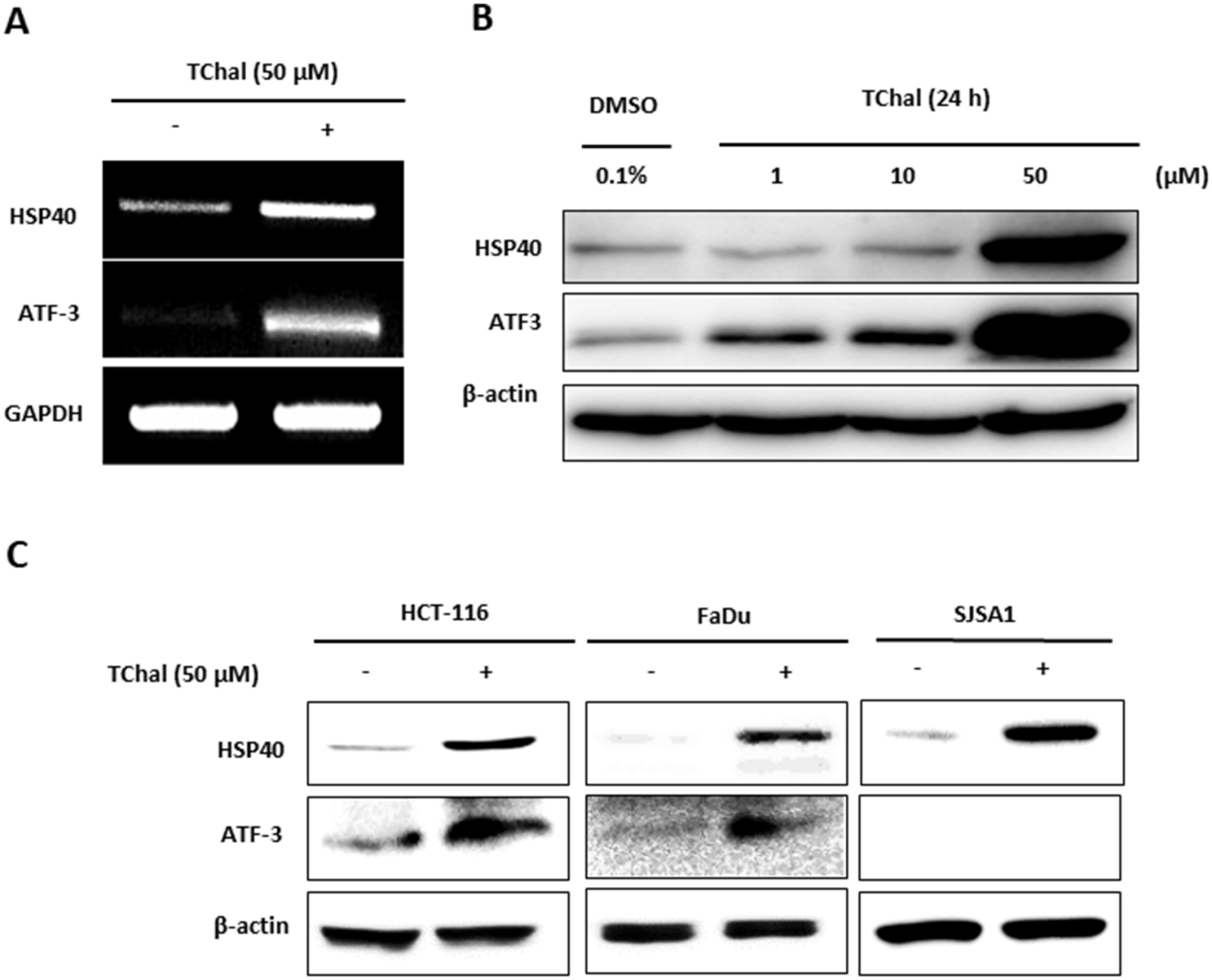
TChal induced Hsp40 and ATF3 expression in cancer cells. **A**. U2OS cells were treated with DMSO or TChal for 24 h. After RNA isolation, RT-PCR was performed using primers for Hsp40 and ATF3, as well as the housekeeping gene, GAPDH. **B**. Western blot analysis of Hsp40 and ATF3 protein expression after TChal treatment. U2OS cells were treated with TChal at the indicated doses, and then cell lysates were subjected to Western blot analysis. **C**. Protein expression of Hsp40 and ATF3 in different cancer cells. HCT-116 (colorectal cancer), FaDu (oral cancer), and SJSA1 (bone cancer) were treated with DMSO or 50 μM of TChal for 24 h. Cell lysates were then subjected to Western blot analysis. A representative gel from 3 independent experiments are shown.

We confirmed that TChal induced the top 2 most induced genes, *HSP40* and *ATF3*, at the transcriptional and translational level using RT-PCR and western blot analysis, respectively (Fig 2A and 2B). As shown in Fig 2B, HSP40 and ATF3 were upregulated following TChal treatment in a dose-dependent manner. To examine whether TChal modified the expression of HSP40 and ATF3 in other cancer cells, HCT-116 (colorectal cancer), FaDu (oral cancer), and SJSA1 (osteosarcoma) cells were treated with TChal at 50 μM for 24 h. The TChal treatment consistently upregulated HSP40 in all the tested cells, and ATF3 in HCT-116 and FaDu cells (Fig 2C). We were unable to detect ATF3 in the SJSA1 cells. These results suggested that TChal upregulated the *HSP40* and *ATF3* transcripts, thereby increasing the expression of the protein levels. The fact that TChal increased HSP40 expression in both the mRNA and protein levels in U2OS cells, and in the protein levels for all the other tested cancer cells, prompted us to characterize HSP40 as a molecular target of TChal in anti-tumorigenesis.

### Chalcone derivatives increase HSP40 in osteosarcoma cells

**Fig 3:**
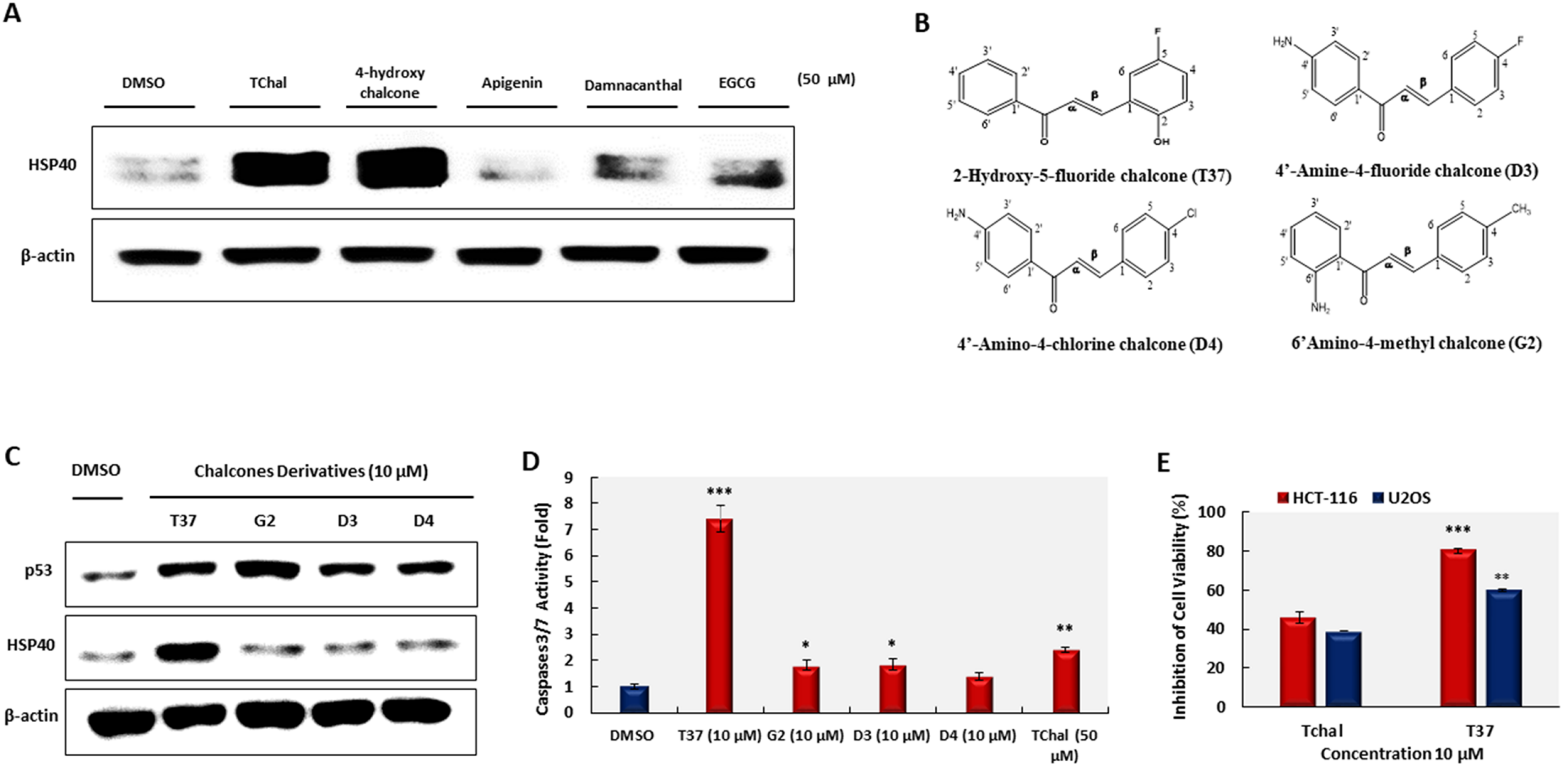
Effect of chalcone derivatives on p53 and Hsp40 protein expression, cell growth, and caspase-3/7 activity. **A**. Western blot analysis of HSP40 protein expression after treatment with 50 μM of different compounds for 24 h. A representative gel from 3 independent experiments are shown. **B**. Structure of chalcone derivatives. These figures were drawn using ChemDraw Ultra 8.0. **C**. Protein levels of p53 and HSP40 after treatment with chalcone derivatives. U2OS cells were treated with derivatives at the indicated doses for 24 h, and then cell lysates were subjected to Western blotting. A representative gel from 3 independent experiments are shown. **D**. U2OS cells were treated with TChal (50 μM) or chalcone derivatives (10 μM) for 24 h. Caspase-3/7 activity using 30 μg of cell lysate was measured using the Apo-ONE Homogenous Caspase-Glo 3/7 Assay kit. Values are expressed as mean ± SD of three replicates. *, *P* ≤ 0.05; **, *P* ≤ 0.01; and ***, *P* ≤ 0.001 versus DMSO-treated cells. **E**. U2OS cells were treated with DMSO (0.1%) or 10 μM of TChal and T37 for 24 h, and then cell growth was measured via MTS assay. Values are expressed as mean ± SD of four replicates. **, *P* ≤ 0.01 and ***, *P* ≤ 0.001 versus TChal-treated cells.

Since chalcone produced by chalcone synthase is a key molecule that is converted into several phytochemicals in plants [23], we compared the effect of TChal on the levels of HSP40 expression with 4-hydroxychalcone and other phytochemicals, apigenin, damnacanthal, and EGCG (identified as a p53 inducer) [19, 24, 25]. The TChal and 4-hydroxychalcone treatments strongly increased HSP40; however, they either poorly induced or did not induce HSP40 protein levels in U2OS cells (Fig 3A). These results suggest that the chalcone’s backbone structure possibly plays a major role in HSP40 induction. We subsequently synthesized 4 different TChal based on the Claisen-Schmidt condensation reaction [26] between acetophenone derivatives and aromatic aldehydes, using green solvents and catalysts (Fig 3B). These derivatives were tested for the expression of HSP40 and p53, and to induce apoptosis via caspase activity. As shown in Fig 3C, all the derivatives highly induced p53; however, HSP40 induction was seen only in T37-treated cells. Moreover, T37 exhibited the highest caspase activity when compared to other chalcones (Fig 3D), suggesting that both p53 and HSP40 induction are critical in increasing caspase activity in U2OS cells. Furthermore, T37 treatment resulted in reduced cell viability in two different cells (HCT-116 and U2OS), with better inhibition (Fig 3E) when compared to that of TChal-treated cells. This result indicates that substitutions of the TChal compound may increase biological activity and provide an excellent candidate compound to develop better anti-cancer compounds.

### HSP40 participates in the stabilization of p53 protein mediated by TChal

**Fig 4:**
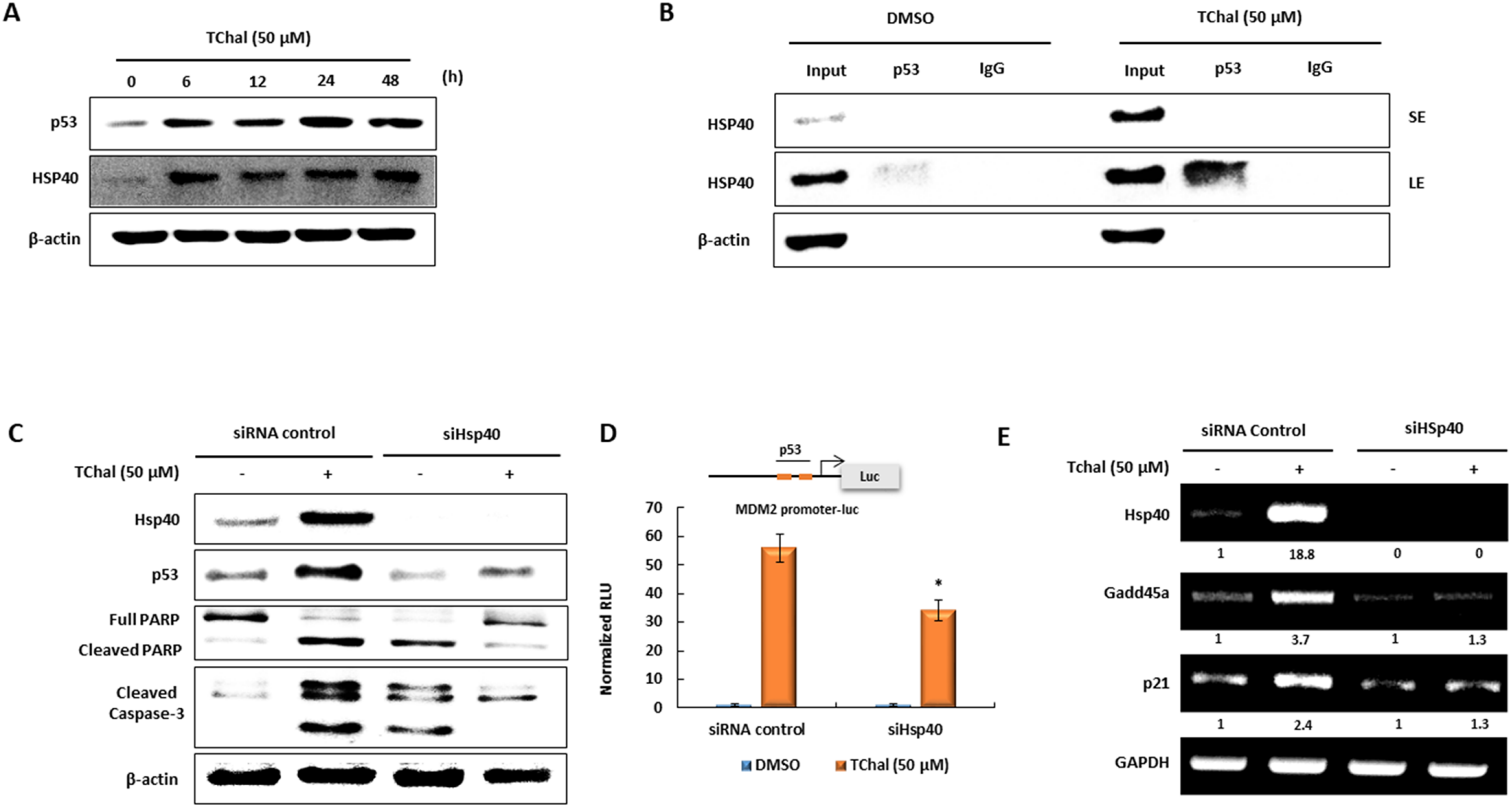
The induction of HSP40 by TChal contributes to the stabilization of p53 activity. **A**. U2OS cells were treated with TChal at the indicated dose and times. The p53 and HSP40 expressions were measured by western blot analysis. **B**. U2OS cells were treated with 50 μM of TChal for 24 h, and cell lysates were isolated with a modified RIPA buffer. The cell lysates were immunoprecipitated with 2 μg of p53 or normal IgG antibodies using A/G PLUS-agarose, as described in the Materials and Methods section, and then subjected to western blot analysis using Hsp40 antibody. **C**. U2OS cells were transfected with 30 nM of Hsp40 or control siRNAs for 24 h, followed by treatment with 50 μM of TChal for 24 h. Then, cells lysates were subjected to western blot analysis. **D**. Vectors containing the MDM2-Luc promoter were co-transfected with Hsp40 or control siRNAs into U2OS cells, and cells were treated with 50 μM of TChal for 24 h. Luciferase activity was measured; y-axis represents RLU (relative luciferase unit). **E**. HSP40 silencing suppressed the effect of TChal on p53-dependent transcription. U2OS cells were transfected with Hsp40 or control siRNAs, followed by treatment with 50 μM of TChal for 24 h. Total RNA was isolated, and RT-PCR was performed as described in the Materials and Methods section. A representative gel from 3 independent experiments are shown.

It has been reported that HSP40 is able to bind to wild-type p53 and stabilize its active conformation [27]. Therefore, we assessed whether HSP40 induction influenced the stabilization of p53 promoted by TChal. First, we examined if HSP40 and p53 were concomitantly expressed in the presence of TChal. HSP40 and p53 were induced as early as 6 h in the presence of TChal (Fig 4A), indicating the possible participation of HSP40 in p53 stabilization. We further investigated the interaction between HSP40 and p53 via immunoprecipitation. As expected, p53 binding to HSP40 was strongly enhanced by TChal (Fig 4B). Furthermore, U2OS cells were transfected with siRNA against *HSP40*, followed by treatment with TChal. As shown in Fig 4C, increasing p53 expression via TChal resulted in the increase of apoptosis as assessed by PARP and caspase-3 cleavage (Fig 4C, lane 1 and 2). However, the silencing of *HSP40* partially blocked p53 upregulation induced by TChal, indicating that TChal-mediated HSP40 induction was mediated by p53 activation in U2OS cells. HSP40-silencing also inhibited PARP and caspase-3 cleavage induced by TChal, suggesting that HSP40 upregulation played a role, at least in part, in TChal-mediated apoptosis induction (Fig 4C). We also examined whether HSP40 influenced p53 activity in transcriptional regulation. An MDM2 promoter containing p53 binding sites was co-transfected with either siRNA control or siRNA *HSP40* and transferred into U2OS cells. As shown in Fig 4D, the induction of MDM2 promoter activity, caused by TChal, was partially, but significantly decreased by HSP40 silencing. This result is confirmed by RT-PCR experiments evaluating the expression of p53 target genes. As shown in Fig 4E, *HSP40* silencing blocked the upregulation of *p21* and *Gadd45a* mediated by TChal, suggesting that HSP40 induction by TChal clearly contributed to p53 activity, probably mediated by p53 stability.

### TChal interferes with p53 and CRM1 interaction

**Fig 5:**
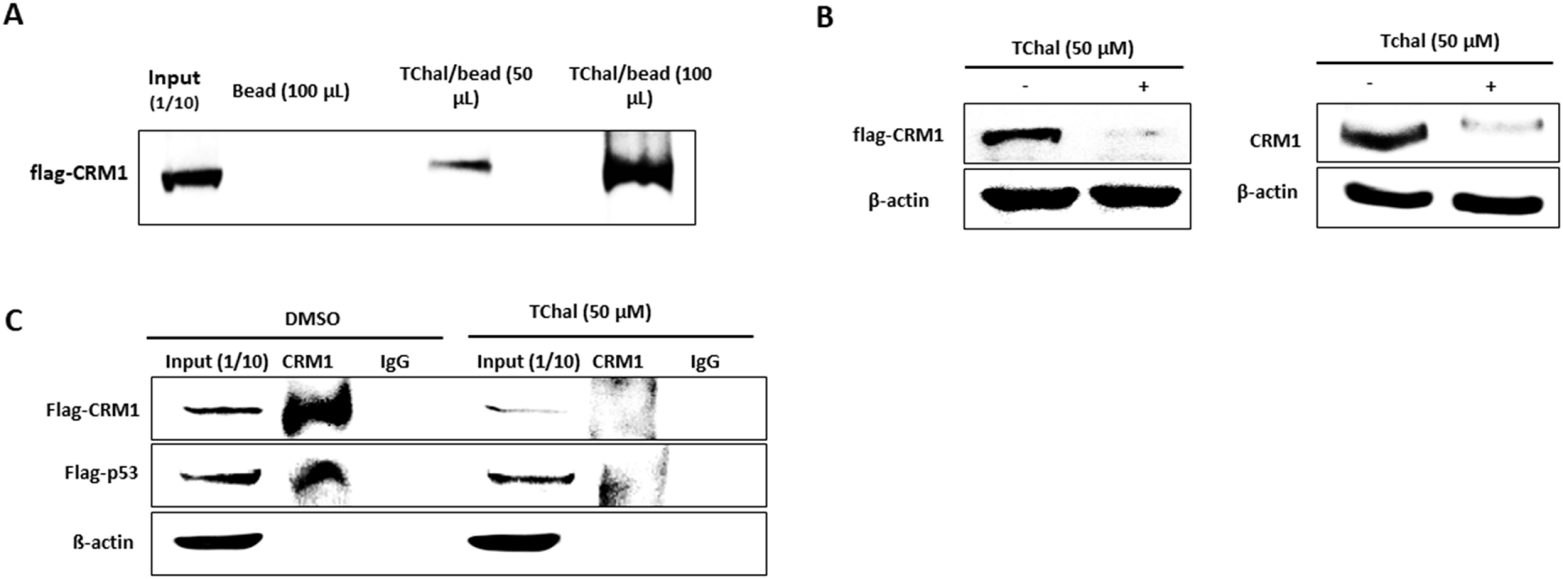
TChal regulates the translocation of p53 via CRM1 downregulation. **A**. Free-magnetic beads or beads bound with TChal were obtained as described in the Materials and Methods section, and incubated overnight at 4 °C with cell lysates from U2OS cells overexpressing CRM1. The beads were washed and collected, and then subjected to Western blot analysis. **B**. Cells transfected with 2.5 μg of Flag-CRM1 vector were treated with 50 μM of TChal for 24 h. The cells lysates were obtained and subjected to western blotting. Left, Western blot using Flag antibody; Right, Western blot using CRM1 antibody. **C**. U2OS cells were co-transfected with 1.25 μg of Flag-CRM1 and Flag-p53 vectors, followed by treatment with TChal for 24 h. Cell lysates were isolated using modified RIPA buffer, immunoprecipitated with 2 μg of CRM1 or normal IgG antibodies as described in the Materials and Methods section, and then subjected to Western blot analysis using Flag antibody. A representative gel from 3 independent experiments are shown.

CRM1 dysregulation in cancer cells plays a role in the translocation of nuclear proteins out of the nucleus. For example, exportation of p53, p21, p27, APC, HSP40, and NAG-1 to the cytoplasm is mediated by CRM1, and their activities can be altered in case of CRM1 dysregulation [17, 28]. Moreover, several phytochemicals inhibit CRM1 activity [29]. Therefore, we assessed whether TChal could regulate the translocation of p53 by acting on the CRM1 protein. A synthetic CRM1 inhibitor, Leptomycin B, has been known to directly bind to the CRM1 protein [30]. We first tested whether TChal was able to bind to CRM1. TChal successfully conjugated with the magnetic beads and incubated with cell lysates overexpressing Flag-CRM1. As shown in Fig 5A, increasing amounts of Flag-CRM1 were precipitated when more TChal/beads were incubated with cell lysates, indicating that TChal was capable of binding to CRM1 in a dose-dependent manner. Selective inhibitors of nuclear export (SINE) have also been reported to bind to CRM1 and attenuate CRM1 expression [31]. Therefore, we determined whether TChal degraded CRM1 expression in U2OS cells. Western blot analysis showed that TChal greatly reduced the levels of endogenous and exogenous CRM1 protein (Fig 5B), suggesting that TChal could be a SINE. Finally, we examined the interaction between p53 and CRM1 in the presence of TChal. U2OS cells were co-transfected with Flag-p53 and Flag-CRM1, followed by treatment with TChal. Cell lysates were immunoprecipitated using the CRM1 antibody. As expected, treatment with TChal decreased CRM1 levels, and accordingly reduced the amount of p53 protein bound to CRM1 (Fig 5C). Cumulatively, TChal not only degraded CRM1 expression, but also interfered with the interaction between p53 and CRM1.

### TChal affects CRM1 and accumulates tumor suppressor proteins p53 and NAG-1 in the nucleus

**Fig 6:**
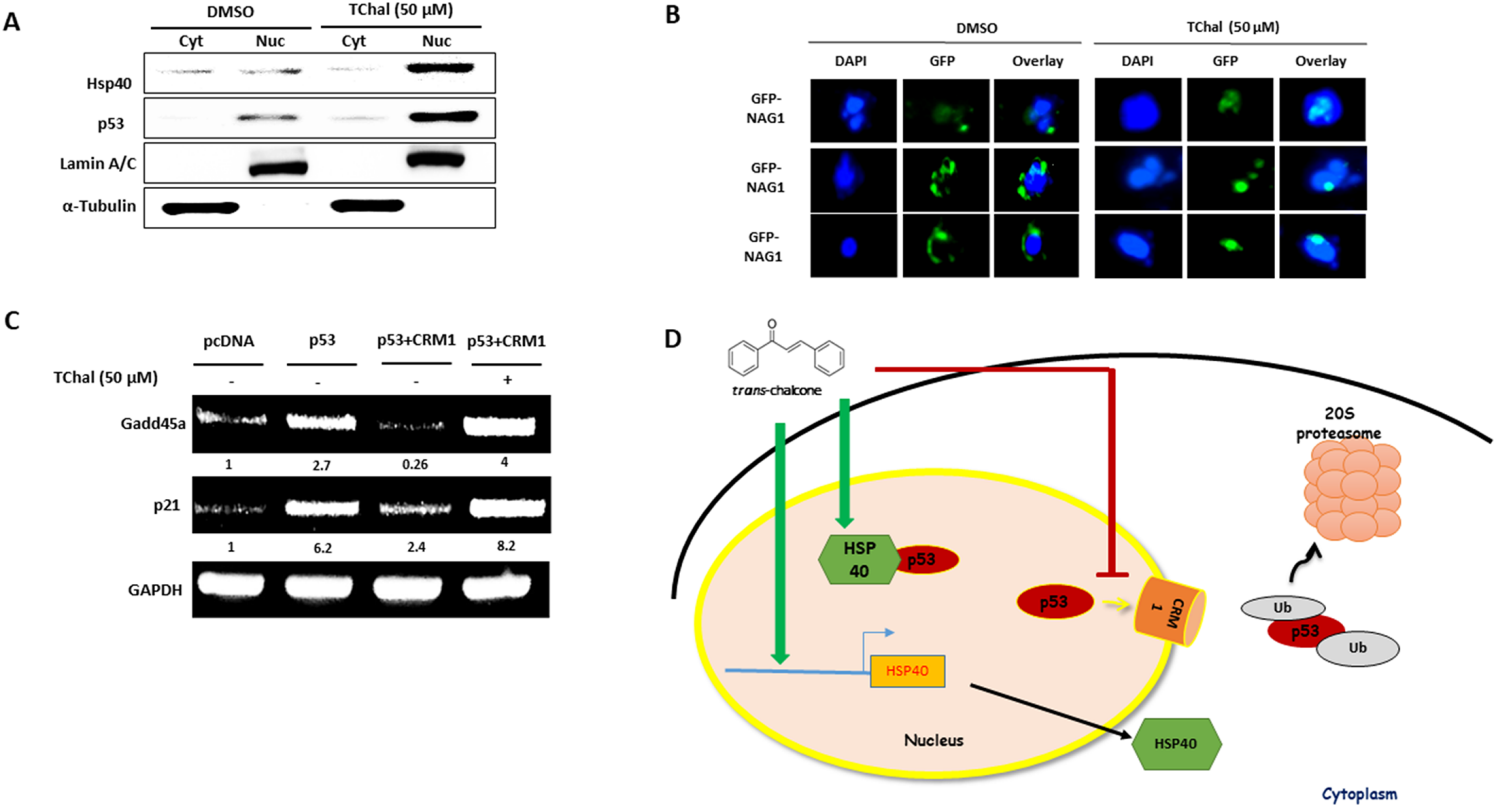
TChal regulates p53 activity via CRM1 inhibition. **A**. U2OS cells were co-transfected with 2 μg of Flag-CRM1 and 0.4 μg of Flag-p53 vectors, followed by treatment with 50 μM of TChal for 24 h. The cytoplasmic (Cyt) and nuclear (Nuc) fractions were obtained and subjected to western blot analysis using Flag antibodies. A representative gel from 3 independent experiments are shown. **B**. U2OS cells, transfected with 2.5 μg of GFP-tagged NAG-1 (WT) and treated with TChal for 24 h, were fixed and analyzed by fluorescence using the EVOS FL Auto Cell Imaging System. DAPI was used to stain the nuclei. Three independent fields are shown. **C**. U2OS cells were transfected with 0.4 μg of Flag-p53 vector, or co-transfected with 0.4 μg of Flag-p53 and 2 μg of Flag-CRM1 vectors, and then treated with 50 μM of TChal for 24 h. Total RNA was isolated, and RT-PCR was performed as described in the Materials and Methods section. A representative gel from 3 independent experiments are shown. **D**. A proposed model for *trans*-chalcone’s action on the stabilization, translocation, and activity of the p53 protein. *Trans*-chalcone increases the expression of HSP40 protein, which stabilizes the native conformation of the p53 protein. *Trans*-chalcone interacts with and decreases CRM1 expression, promoting the accumulation of p53 into the nucleus, and thus increasing the transcriptional activity of p53.

To further investigate the effect of TChal on the translocation of p53, HSP40, and NAG-1, expression of endogenous p53 and exogenous NAG-1 was examined. As shown in Fig 6A, TChal treatment resulted in the accumulation of HSP40 and p53 in the nucleus. Immunohistochemistry analysis also indicated that NAG-1 accumulated in the nucleus in the presence of TChal (Fig 6B). To examine whether TChal was able to increase p53 activity even in the presence of high amounts of CRM1, cells were transfected with p53 and CRM1, followed by treatment with TChal. Two p53 target genes were investigated. As shown in Fig 6C, overexpression of p53 increased *Gadd45a* and *p21* transcript levels; however, CRM1 overexpression with p53 significantly suppressed *p21* and *Gadd45a* induction, which was mediated by p53. This result suggested that even high amounts of p53 may be ineffective at inducing p53 target genes when CRM1 is overexpressed. Interestingly, treatment with TChal inhibited the suppression caused by CRM1 overexpression on the induction of *p21* and *Gadd45a*, indicating that possibly TChal could increase p53 activity by inhibiting CRM1.

## Discussion

The p53 tumor suppressor protein is a moonlighting protein that plays different roles in different cellular locations. For example, p53 acts as a transcriptional factor in the nucleus; however, in the mitochondria, p53 has a role as an apoptosis effector. Thus, controlling translocation of p53 in the cells is important for its activity. It is tightly regulated by several factors including nuclear exporters and chaperone proteins that possibly control the translocation mechanism.

Heat shock proteins (HSPs) are other moonlighting proteins that play important roles in the unfolded protein response. HSP dysregulation is implicated in a variety of human diseases, including cancer. Among those, DnaJ/HSP40 proteins are well-known co-chaperones that regulate the ATP hydrolysis activity of HSP70 [32]. However, research has revealed that they function as independent chaperones, particularly in various types of cancers [33]. Although the exact role of DnaJ/HSP40 proteins and the molecular mechanisms by which they are involved in cancer biology need further investigation, recent studies have provided critical clues. It appears that the DnaJ/HSP40 protein family is involved in cancer development by directly regulating the stability of proteins such as tumor suppressors [34].

Flavonoids have been extensively investigated as potentially useful compounds in the treatment of various diseases. Interestingly, TChal and its derivatives exhibit intense anti-cancer activities in various cancer cell lines. One interesting feature of TChal is that it does not exhibit genotoxic effects on the amino group of nucleic acids, which most anti-cancer drugs were found to possess. Moreover, we recently demonstrated that TChal exhibited anti-cancer activities, which was mediated by an increase of the tumor suppressor protein, p53 [21]. Chalcone derivatives with different chemical structures in the p53 pathway have been linked to mechanisms such as the disruption of p53/MDM2 interaction, decrease in p53 ubiquitination, inhibition of the chymotrypsin-like activity of the 20S proteasome, and induction of SIRT1-mediated hyperacetylation of p53 [35–38].

In this study, we found that TChal increased HSP40, so as to make it the most highly expressed gene in U2OS cells. Increasing HSP40 causes PARP and Caspase 3 cleavage (Fig 3D). However, the silencing of HSP40 induces moderate cleavage of PARP and caspase 3 (Fig 4C). It is likely that p53 may be trapped in the cytoplasm and be prevented its translocation to the nucleus through PDCD5 [39], resulting in increasing caspase cleavage. Furthermore, to examine the effect of TChal on DNA damage markers, p-H2AX was investigated and found that TChal do not alter p-H2AX expression (data not shown), suggesting that TChal induces p53 expression via HSP40 and CRM1. As a consequence, RNA-Seq data suggested that TChal induced the expression of several genes categorized as positive regulators of apoptosis, with many genes described as p53 target genes. The previous studies also indicated that HSP could be regulated by a variety of flavonoids. For instance, the regulatory effects of fisetin on HSP70 [40], quercetin on HSP27 and HSP70 [41], and luteolin on HSP90 [42] were previously documented. Recently, it was also reported that luteolin increased HSP40 as the most highly induced gene, raising the possibility that HSP40 might play a regulatory role in anti-tumorigenesis [43].

The chemical structure of TChal is based on a common 1,3-diphenyl, propenone template that can be easily modified by the addition of a wide variety of functional groups (aryls, halogens, hydroxyls, carboxylic groups, benzenes, etc.), resulting in alterations of their biological activity. In order to explore new TChal derivatives, compounds were synthesized using the Claisen-Schmidt condensation reaction (Fig 4). Interestingly, T37 exhibits better activity in terms of apoptosis and cell growth inhibition. This compound, compared to others, does not possess amine groups in its structure. Further investigation is needed to elucidate how this amine group contributes to the biological activity. The structure of the compounds was confirmed by ^1^H and ^13^C Nuclear Magnetic Resonance (NMR) and bi-dimensional NMR techniques.

CRM1 is a nuclear export receptor involved in the active transport of tumor suppressors. Its function is altered in cancer due to increased expression and overactive transport. CRM1 could be considered a promising therapeutic target for anticancer drug development because overexpression of CRM1 has been correlated with poor prognosis of patients in several cancers. Tumor suppressor proteins p21, p73, APC, NAG-1, and p53 play a pivotal role in tumorigenesis and are known to be translocated in the cells via CRM1 [9, 17]. In this report, we demonstrated that TChal binds to CRM1 and inhibits its activity. TChal also disrupted the interaction between tumor suppressors such as NAG-1, p53, or p21, and CRM1, thereby inhibiting the export of these proteins to the cytoplasm. We further determined if TChal bound to the sulfhydryl group of Cys^528^ in CRM1 as previously shown with leptomycin B [30]; however, TChal was found to not bind to the same site on CRM1 as leptomycin B (data not shown). Since the clinical development of leptomycin B has been limited by its toxicity and narrow therapeutic window in preclinical animal models, orally bioavailable small-molecule selective inhibitors of nuclear export (SINEs) that specifically and irreversibly bind to residue Cys^528^ in the cargo-binding groove of CRM1 have been developed and tested with positive outcomes [44]. Thus, TChal may be another CRM1 inhibitor that exhibits anti-CRM1 activity with less side effects.

Overall, we found that TChal binds and degrades CRM1, and increases HSP40 expression, thereby enhancing p53 and other tumor suppressor protein activities in cancer cells (Fig 6D).

## Supporting information

S1 Table Raw data for up- and down-regulated genes by trans-chalcone. (XLSX)
